# Surgery

**Published:** 1836-04

**Authors:** 


### SURGERY.

### *On the Nature and Treatment of the Vertebral Disease of Pott.* By M. Richet, first Surgeon at the Hôpital de la Charité at Lyon.

The present essay consists of a well-collected series of cases illustrative of the vertebral disease of Pott, the appearances of which, on examination after death, are very minutely detailed. We must limit our extracts to the conclusions which M. Nisbet has drawn from these cases, in which he endeavours to establish the fact that the spinal disease bearing the name of Pott is tubercular, and to show that hitherto a partial or incorrect view has been taken of its pathology. We may select the following example from his numerous cases, as exhibiting the chief peculiarities of the morbid anatomy of this affection.

A man, aged 20, two years previous to his admission to the hospital, had been affected with a painful swelling between the last dorsal and first lumbar vertebræ. There was great feebleness, but perfect sensation in the lower extremities. Slight but temporary relief followed the application of cauteries to the swelling: the paralysis returned. The lower extremities became œdematous; ascites; commencing gangrene about the right spine of the ilium; increased pain in the loins, with complete paralysis of the lower limbs, terminating in death, five months after the admission of the patient.

*Dissection.* The anterior common ligament in front of the bodies of the last five dorsal and first two lumbar vertebræ was elevated an inch, by thick, yellow, viscid, tubercular matter. The first lumbar vertebra was destroyed, excepting a small fragment attached to the healthy intervertebral cartilage of the last dorsal vertebra. The second lumbar vertebra was reduced to half its thickness; the last five dorsal vertebræ were filled with crude tubercles, their cavities communicating in front with the tubercular cyst, and with one another in the bodies of the vertebræ. From two vertebræ the matter had escaped into the spinal canal, and had depressed the posterior common ligament, the membranes, and the chord. Tubercular matter had infiltrated the seventh dorsal vertebra; the body of the bone being easily cut, and the surface of the incision of a yellowish white colour. The five last vertebræ, freed from their tubercular matter, appeared rough and like shagreen; their surfaces hollowed, and the concavities subdivided by sharp, longitudinal partitions, some of which were thin and full of apertures. Their texture was however hard, and sounded when struck in a manner quite different from a carious bone. A thin cellulo-vascular membrane, of a deep red colour, lined their cavities. The back part of the third lumbar vertebra contained a large cavity full of tubercular matter, which also filled the corresponding part of the vertebral canal, pressing backwards the dura mater. From this point to the middle of the back, the dura mater and arachnoid were of the colour of the dregs of wine; the spinal marrow was sound. The intervertebral cartilages were healthy. The ribs corresponding to the diseased vertebræ were soft, flexible, and contained a dark fluid. Tubercular matter, in smooth cysts, was deposited in the neighbouring parts, and particularly in the left iliacus muscle, having no connexion with the disease of the vertebral column. Both lungs were filled with miliary tubercles.

The scrofulous tubercle, which M. Nisbet found to exist in all the cases of the vertebral disease of Pott which have fallen under his own observation, he considers as the origin of the affection. It appears under various forms, and in different situations: 1, as single or several deposits, varying in size from that of a pea to that of a nut; of a white, yellow, or grey colour; in cavities, either isolated or communicating with one another, either opening anteriorly or into the vertebral canal: 2, more rarely infiltrated into the spongy tissue of the bones, the texture of which has undergone no other change than a diminution of consistence: 3, amorphous masses of tuberculous matter, of various size, frequently deposited beneath, and elevating the anterior and posterior common ligaments.

The dorsal is more frequently the seat of these tubercular deposits than either the cervical or lumbar regions. Most commonly the tubercles are deposited in the bodies of the vertebræ. It is rare to find them confined to one bone. Any portion of the atlas and axis may be the nucleus of tubercles, the consequent phenomena being very different from those observed in the other situations of the same disease. Tubercles sometimes originate within the intervertebral cartilages, which may be affected alone or simultaneously with the vertebræ. In every situation, their first effect is to hollow a cavity for their reception. The bony parietes of these cavities preserve their normal structure and consistence, their production being apparently the effect of simple absorption from pressure, without inflammation. When the tubercles are not in immediate contact with the bone, their cavities are lined with a thin, vascular false membrane; and this is generally accompanied by a smoothness of the adjoining portions of bone. Tubercles formed in the centre of the bodies of the vertebræ gradually destroy them, until a kind of shell is all which remains: being no longer able to sustain the weight to which it is subjected, this breaks, and the column inclines, allowing the opposite bones to come in contact. If the tubercular matter be deposited within the bone, in cavities separated by thin septæ, these for a time afford support, but they eventually give way; and the inclination occurs somewhat suddenly, and at the moment of the destruction of these partitions. When the tubercles are first formed on the anterior surfaces of the bodies of the vertebræ, the destruction takes place slowly backwards; deformity occurs more gradually, and, as several vertebræ are generally implicated in the disease, instead of the angular prominence caused by the obliteration of a single bone, the spine assumes the form of a curve. It is rare to find tubercles deposited only behind the bodies of the vertebræ, but trifling protuberance results; the effect being chiefly confined to compression of the chord, and consequent paralysis. Lateral destruction of the vertebræ produces a corresponding deformity. The small extent of bony matter in the atlas and axis is rapidly destroyed by tubercles; the head inclines towards the part which is deficient, the ligaments are stretched or torn, and a fatal termination results from compression of the chord. The processus dentatus, when partially absorbed, may be separated from its connexions by a sudden motion of the head, and produce death by pressure on the chord. An ulceration of the articular cartilages of the first two vertebræ gives rise to elongation of their ligaments; the atlas slides downwards and forwards, its posterior arch compressing the chord; the movement of the dentata backwards contributes to the same result; paralysis gradually increases, and death supervenes by asphyxia. In an advanced stage, the intervertebral cartilages of these bones become softened and absorbed. It may happen that, by the loss of a body of a vertebra, two cartilages come in contact, and are then rubbed away. More rarely, tubercles are deposited within the substance of cartilages, which are then destroyed by ulceration. Paralysis does not always accompany this vertebral disease: when it does occur, it is limited to the organs inferior to the spinal lesion. Its forms are, simple loss of power, associated or not with loss of sensation; permanent contraction of the muscles, with or without sensibility, or accompanied by acute pains in the contracted limbs. The curvature of the spinal column does not necessarily produce paralysis. The spontaneous luxation of the atlas appears to be the only instance of displacement of a vertebra causing paralysis. In the majority of cases where paralysis has existed, its cause, on examination after death, has appeared to be, 1, an effusion of tubercular matter between the meninges of the chord; 2, tubercular matter within the chord, which has been found completely divided, *(Gazette Méd.* 1830;) 3, tubercles in the meninges, either infiltrated or deposited in small masses and sometimes coalescing, accompanied by thickening of the membrane, *(Ollivier, Maladies de la Moelle Epinière;)* 4, slight or extensive softening; or, 5, induration of the chord; 6, thickening and injection of the dura mater and arachnoid; 7, bony spiculæ penetrating the membranes.

These various conditions either compress, irritate, or give rise to inflammation of the chord. An exact relation exists between the different forms of paralysis and the organic lesion. With very considerable compression of the chord, with an interruption of its continuity, and with its softening, are associated complete loss of motion and sensation. Paralysis with contraction coincides with inflammation, kept up by some irritant cause; as bony concretions. Loss of muscular contractility alone, with relaxation, depends on a slightly irritating cause. The increase of this cause destroys sensibility; the employment of means which diminish irritation is attended with a return of power and sensation. The earlier existence of the disease in the bodies of the vertebræ, explains the prior affection of muscular contractility, the anterior nerves of the chord being first implicated. The causes of the paralysis explain the utility or inutility of remedies.

The first effect of tubercles on the bone is simple absorption; the texture of the bone around remaining quite normal. At the surface of the loss of substance, the bone may be smooth or rough; of natural colour or slightly injected. The tubercles, in coalescing, isolate various laminæ of bony tissue; which are found dry and mingled with tubercular matter. A single sequestrum of various size is sometimes all which remains of an entire vertebra. Similar destruction is caused by tubercular infiltration. The presence of tubercles sometimes excites a process of reparation; the effect of which is, the formation of bony spiculæ, extending from one vertebra to another; by which these bones may be fixed and a limit put to their destruction. The anterior common ligament is the origin of these new bony formations. Absolute and long continued repose may favour a union of vertebræ partially destroyed, by means of an intermediate cartilaginous deposit, which prevents the motion of the column, and opposes the progress of curvature. But much more commonly the bones are extensively destroyed, in consequence of the constant rubbing of their exposed surface.

The various changes met with in the vertebræ, comprise loss of substance; injection; necrosis; an ivory hardness of bone, and the production of new osseous matter. Swelling, softening, and caries, so commonly spoken of, have never been observed; and it hence appears probable that they have been admitted to exist on insufficient grounds. The deposits of tubercular matter in front of the bodies of the vertebræ have been confounded with swelling and softening of their substance; and Pott, who at first declared himself in favour of the latter opinion, afterwards remarked that "the bodies of the vertebræ were always in a condition which consisted rather in corrosion than in augmentation of volume." In later times, this affection has been attributed to osteitis; but on this supposition, can we explain the occurrence of death with but a superficial destruction of the vertebræ, whilst caries of the whole body often terminates in cure. In Pott's disease, the patients generally die of some other affection. Admitting this supposed cause, we should expect to find the vertebræ softened and suppurating; but this condition, which is not uncommonly met with in the articulating extremities of long bones, is very rare in the spine, and then, apparently, superadded to the other disease. The sequestra in Pott's disease are smooth, white, and of ivory hardness; in inflammation of long bones, terminating by necrosis, they are uneven and angular, fragile and spongy. In the former case, the bone possesses its normal structure; in the latter, it has been softened and expanded by inflammation, previous to its loss of vitality. Caries furnishes an effusion of the colour of wine dregs; softened tubercular matter constitutes the effusion in Pott's disease. Hardness and sonoreity characterise the bone around the tubercular deposits; softness and dulness on percussion, the bone affected with caries. Pott's disease is complicated with various affections. Nine tenths of the cases observed had pulmonary tubercles, and died from this cause; the same deposits may be found in all parts of the system. These tubercles may give rise to abscesses quite unconnected with the vertebral disease. Tubercular deposits, followed by abscesses, and situated in front of the spine, may create suspicion of vertebral disease, when the vertebræ are entirely free from all lesion. A softened mass of tubercular matter has been mistaken for an abscess depending on caries and in process of cure.

*Treatment.* When there is much pain in the protuberance, at the base of the thorax, in the epigastrium or loins; local bleedings are useful. In all the cases in which pain (which is not a constant attendant on disease of the spine,) has been complained of, the chord or its membranes have been found variously inflamed; and it is by remedying this condition, that such means are useful. Cauterization is not long before it produces relief of pain: respiration and the movements of the trunk become easier; there is some return of power and sensation of the lower limbs, and the amelioration may proceed still further, until the patient can take considerable active exercise, even whilst the protuberance remains. When, after death from other causes, the body has been examined in this state, the vertebræ have been found tubercular, eroded, hard, and like ivory; the continuity of the column has been interrupted at the situation of the protuberance, and there has been no attempt at reunion; some slight inflammatory appearances of the meninges or chord have remained, the consequences of a condition, which the cauterization had relieved. The pains and paralysis had diminished, together with the inflammation. When the pain and paralysis have obstinately resisted cauterization, the spinal inflammation has been found dependent on an immoveable and active cause. The progress of the vertebral disease is then quite uninfluenced by emunctories; it is always proportional to the number of the tubercles and to the movements of the trunk.

The movements and weight of the body hasten the softening of the tubercles, wear away the bodies of the weakened and almost destroyed vertebræ, give rise to friction of contiguous bony surfaces, irritate the chord and cause paralysis, promote the formation of abscesses by exciting inflammation of the soft parts, tend to demolish the bony columns which may be formed, and if the disease be situated in the first two vertebræ, produce a sudden and fatal compression. Hence, as remedial means, the necessity of repose and the horizontal position.

*Gazette Médicale de Paris*, Nos. 34 and 34. Août. 1835.

---

*Treatment of Scald Head, (Teigne, Fr. Porrigo.)* By Baron Alibert.

The hair is cut as close as possible, and the head is washed for some time with a solution of carbonate of soda, or infusion of walnut leaves. An ointment, containing one drachm of soda to an ounce of lard, or two drachms of soda if the hair is thick, is then rubbed in, and the head covered with a piece of blotting paper. Mild bitter infusions are given internally; and, if it is suspected that the parents have a syphilitic taint, antiscorbutic medicines with mild mercurials. If the soda is inactive, it may be replaced by potash. In some cases, an ointment made with the burned leaves of belladonna or stramonium, or the same mixed with water, removes the disease.

[Those affected with scald head in the dispensaries and hospitals of Paris have been put by government under the care of two brothers, named Mahon, who have cured above 50,000 cases in these public establishments. They begin by cutting the hair short, (to within two inches of the scalp,) and, after removing the scabs with emollient poultices, they wash the head with soap and water for several days, until it is very clean. They then apply a depilatory ointment, which removes the hair slowly and without pain, and sprinkle the head with a powder which forms the basis of the ointment, taking care to keep the scalp very clean. The composition of this ointment they keep secret. M. Rayer has endeavoured to supply its place with one consisting of an ounce of prepared chalk, two drachms of subcarbonate of potash, and one drachm of powdered carbon, mixed with lard; the quantity of which is to be increased or diminished in proportion as the scalp is more or less inflamed. M. Biett often prescribes the following lotion instead of the depilatory powder:

℞. Sodæ Sulphur. iij.; Saponis Castil. ℥ss.; Alcohol, ʒij.; Aquæ Calcis. lbj.

Misce, fiat lotio.

M. Giscard, an army surgeon, employs successfully this ointment:

℞. Axungiæ, lb. ij.; Sulphuris, ℥ij.; Pulv. Carbonis, ℥viij. M. fiat ung.

After shaving the head, he directs a layer of this ointment to be applied over it, to be washed off two or three days afterwards with a solution of black soap. This application, repeated five or six times, has cured the most complicated cases.]

*Journal de Médecine et de Chirurgie pratiques, Novembre*, 1835.

---

*On Balsam of Copaiba in Gonorrhœa.* By M. Ratier.

M. Ratier adopts the plan of treating gonorrhœa, which is very general in this country, by means of copaiba in the early stages. A modification he advises may be very useful in some cases,—that of exhibiting the balsam as an emulsion in an enema, when it disagrees with the stomach. He finds that the action of copaiba is impaired by any quantity of fluids; he therefore gives it on sugar or in a teaspoonful of wine, and recommends the patient to drink as little as possible. He prescribes a mild and nutritious diet, and forbids stimulants.

*Bulletin général de Thérapeutique, Septembre*, 1835.

---

*On the Use of the Datura Stramonium, in Sarcocele and Diseases of a similar character.* By Thomas T. Everitt, M.D., of Poughkeepsie, N. Y.

Sarcocele is generally regarded as a disease of which the progress and advancement are only to be interrupted, and its evils avoided, by castration. Like most diseases of the glandular system, which are characterized by increase in size, and other evidences of morbid action, without acute inflammation, it has engaged the attention of the surgeon only with a view to an operation, as a measure inseparably connected with the disease, and one that sooner or later, as circumstances dictated, must be resorted to. If attempts have been made heretofore, to retard its development, they have been unsuccessful, so far as can be ascertained; and in the treatment of it, such course in practice usually adopted as would tend to the comfort and convenience of the patient; palliating his distress, and affording momentary relief, but nevertheless, with an assurance which experience has invariably taught, that the knife was the only sure and certain remedy, and to its power the patient must ultimately resign himself.

The partial successful treatment of two cases of sarcocele leads the writer to hope that remedial agents may yet be found, to which enlarged and diseased glands will readily yield. And with the intention of eliciting the attention of the profession to the subject, he is induced to report the results of his efforts.

In two cases of the disease which have been subjected to the use of the stramonium, both have been materially benefited, and in one the morbid action and train of disease entirely arrested; the tumour lessened in size, and most of the attendant evils removed. In connexion with its use the suspensory bandage has been worn, and mild cathartics administered at intervals of eight or ten days. For the period of two months the patient made use of the ointment of stramonium of twice the usual strength, applying it plentifully to the scrotum with considerable friction. At the same time he was directed to take of the extr. stramonii a quarter grain every morning and evening. For a small part of the time pil. hydrarg. was administered, and at different times in the course of the treatment other medicines, as iodine, &c., but none continued for any length of time, and there can be no doubt of the improvement in the case being altogether the effect of stramonium. Having much faith, therefore, in its medical properties, I would recommend its use more generally in affections of the glandular system, believing it will be found useful and efficacious.

*United States Med. and Surg. Journal, for October*, 1835.

---

### *Ointment to allay the Irritation of Hemorrhoidal Tumours.* By E. GEDDINGS, M.D.

So much suffering is experienced from the incessant irritation of hemorrhoidal tumors, that every means capable of affording relief under such circumstances must prove acceptable to the profession. I have used an ointment similar to the following, with the most happy effects, and in a great number of cases:

℞ Pulv. Carb. Plumbi, ℥ ss. Sulph. Morph. gr. xv. Unguent. Stramon. ℥ j. Ol. Olivar. qs. M. ft unguent. part. applicand.

Powdered opium, to the amount of a drachm, may be substituted for the morphia, and if the dry white lead is not at hand, that which is ground in oil, for the use of painters, may be advantageously substituted. Sometimes a drachm of powdered galls may be added. *American Archives*, No. I. Oct. 1834.

---

### *On a new Mode of Treating Burns.* By Professor VELPEAU.

M. VELPEAU recognizes four degrees of burns: in the first there is simply rubefaction; in the second, vesication; in the third, the rete-mucosum and first layer of the true skin are involved; and in the fourth, a part or the whole thickness of the skin is converted into an eschar. If left to themselves, the first either terminates in resolution from the second to the eighth day; or changes into true erysipelas, or vesicates; the second rarely recovers in less than ten or twenty days, as the epidermis becomes detached and suppuration established; the third is followed by ulceration of the dermis, and the cure is delayed for a month or more; the slough is not detached from the fourth in less than ten or twenty days, nor is the ulcer healed under from two to six weeks. The merits of numerous remedies for burns are examined in relation to these degrees.

1. *Cold Water.* This is useful only in the first degree, which it cures in three or four days.

2. *Methodical compression with a Roller.* This is still more useful in the first degree: in the second and third, the epidermis should be removed previously, the wound covered with linen in which numerous small holes have been cut, spread with cerate, and then covered with charpie. It then prevents erysipelas. In the same manner it is useful in the fourth degree.

3. *Cotton, or down of the Typha.* These are useless in the first or fourth: employed early in the second and third degrees, they absorb all moisture, become dry, and a cicatrix forms beneath them, sometimes in a week or fortnight; but very often they form a crust, which does not prevent inflammation or suppuration manifesting themselves. It is a remedy of little value, except in some few situations or cases.

4. *The Solution of the Alkaline Chloride.* This acts much like cold water; but it also improves the state of the ulcerations. M. Velpeau has tried the chlorides of soda and lime in more than fifty cases, but thinks their value has been greatly exaggerated, and that they are merely good detersives.

5. *Saturnine Lotions.* These act in much the same way: they are more sedative and less exciting than chlorides.

6. *Linseed Poultices.* These are too much neglected: they calm irritation, remove the elevated epidermis, and diminish suppuration in young and sanguine habits: where they are not beneficial after a few days, their use should not be persevered in.

7. *Leeches.* These applied around eschars prevent or diminish inflammation and erysipelas.

8. *Liniment of equal parts of Oil and Lime-water.* This in superficial burns, particularly of the face, is very useful; the parts being anointed four or five times daily with a feather. No dressing is required: in five or six days large burns of the first, and some of the second degrees, have been cured by it.

9. *Strapping with Diachylon Plaster.* M. Velpeau originated this plan, and has pursued it with great success since 1832. It fulfils the same indications as compression, besides hastening greatly cicatrization. The first degree is constantly arrested by surrounding the burn in such a manner that the straps will remain seven or eight days, and without hindering the patient from performing his usual duties. In the second and third degree, the epidermis must be first removed, and the wound cleaned, and the strapping renewed every third, fourth, fifth, or sixth day: the cure almost constantly takes place from the fourth to the sixth day, in the second; and from the tenth, fifteenth, or twentieth in the third degree. In the fourth degree it represses the surrounding inflammation; does not hinder the separation of the sloughs, and as they become detached it favours cicatrization. Each strap must be from three quarters to one inch in breadth, and long enough to make one turn and a half around the burned part; the first being placed an inch below, and the last an inch above it: they must overlap each other so that only one-third of each is exposed; they must be very evenly applied, so as to compress equally every part, and the ends must be crossed over the sound skin. If suppuration is profuse, they should be changed every two days, at first: in ordinary cases every three or four days is sufficient. In the fourth degree, before the eschars are detached, the straps should remain for five or six days; the discharge which escapes between them being absorbed by charpie, and the whole surmounted with some compresses and a roller: when changed, the wounds should be cleansed, and even washed with saturnine lotion. No portion of detached epidermis should be left. If the surface of the part burned is uneven, the inequalities must be filled with charpie, or compresses, placed between the strapping and the roller. This treatment is only applicable for the arms and legs, and must be suitably modified for the hands and feet. In very large burns it is not advisable, as the dressing would be inconvenient and difficult. Some skins also will not bear it. The composition of the plaster is important. The linen should be neither too fine nor too coarse; neither too thickly or too thinly covered. That diachylon is best which has less resin and fat and more lead. The effect of this treatment is astonishing. Burns of the limbs generally are cured in three or four dressings. The cicatrix in many respects is remarkable. It forms on all points of the surfaee, denuded to the second degree, at the same time; so that on the first or second removal of the straps it looks large, smooth, and supple, but firm. In the third degree, the same thing is observed: the new skin forming in the centre of the suppurating wound as well as around its edges. [M. Velpeau considers this remarkable, "*une particularité*," but John Hunter has described it as the usual mode in which ulcerations heal where the *whole* thickness of the skin has not been destroyed.] If the ulcer is deeper, its edges soon subside, become white, and then prolonged by a kind of pale thin pellicle over the suppurating surface. The granulations become firm, and are soon replaced by an epidermic surface, even before the bottom of the wound is on a level with its edges, so that the cicatrix remains a long time depressed. As the cicatrix is not formed wholly from the circumference to the centre, and is soft, of equal thickness, and very extensible, it is free from bridles, and irregular and deformed lumps, which follow other modes of cure. As they are less exposed to break and to excoriate, the consecutive retractions are more often avoided.

The duration of the treatment is almost the same in large as in small wounds. When well done, it gives no pain nor irritation: no dressing is so pleasant; and immediate relief follows. As it is changed only every three or four days, it is extremely inexpensive.

The details of sixteen cases are given, illustrating the effects of this treatment, and proving the previous statements. Some of these also prove that cicatrization takes place as rapidly in an ulcer of some standing, as when the surface is first exposed. No other treatment but this is equally suitable in all stages. In order to discover on what the beneficial effects of this treatment depended, M. Velpeau made numerous comparative experiments: thus, to try if the compression was the agent, he tried on the same patient compression by bandages, covering the wound with different dressings, but the burns which were strapped healed more rapidly: he next tried strapping with other plasters, but the diachylon succeeded much more rapidly: finally he compared the effect of covering the burns with disks of diachylon and other plasters without making pressure, and they healed more rapidly under the use of diachylon, but less so than when pressure was used. The union of pressure and the diachylon plaster is therefore essential. It is needless to state that this is merely an application to burns of Baynton's mode of treating ulcers: M. Velpeau has however applied it to ulcerations of all kinds, and even to subcutaneous inflammations and indurations. He proposes to return to this part of his subject at another period.

*Revue Médicale Française et Etrangère, Juin and Juillet*, 1835.

[The above abstract of M. Velpeau's valuable memoir will be appropriately followed by a brief notice of another mode of treating the same lesion, recommended by an American physician, and which we extract from a recent American journal.]

---

### *On the Treatment of Burns with Yellow Wash, (Aqua Phagædenica.)*

### By F. B. E. HINTZE, M.D.

DR. HINTZE, having failed in treating with success by unctuous applications the chronic ulcers which succeed to burns, had recourse to the application of the yellow wash, powdered rhubarb and dry lint, from the known good effect of such applications in other ulcers. The general benefit obtained from this mode of treatment in such chronic cases induced him to employ it in recent burns. He details eight cases in which this plan was successfully followed; but as they present nothing remarkable beyond the result of the treatment, we shall content ourselves with extracting that portion of the paper which contains some general directions for the use of the method recommended.

"The wash," says Dr. Hintze, "which I employ in recent cases, is composed of one grain of corrosive sublimate to an ounce of lime-water. In chronic ulcers, I use it of the strength of one and a fourth grain to the ounce. When called to a case of recent burn, I remove all the vesicles with scissors. I then apply the yellow wash with a soft feather, or a camels' hair pencil, over the whole surface, and dust the part with finely powdered Turkey rhubarb. Over this

I apply soft patent lint, cut into small slips to accommodate them to the part. This is the only local treatment necessary; and, if there should be any indication for constitutional remedies, the means proper in such cases should be employed. Should additional vesicles make their appearance, they must be promptly removed, as the accumulation and detention of a quantity of fluid within them tends to retard the healing process. Where the lint adheres, it should not be disturbed, but any pieces which may become loose may be separated with forceps or scissors. The part should again be wet with the wash, dusted with the powder as before, and covered with fresh lint. The adherent lint should also be moistened with the wash. This process should be repeated at least once or twice a day. Should tension, with more or less thickening, affect the part, generally indicative of the incipient separation of a slough, this process should be promoted by emollient poultices. As soon as the slough has separated, the former remedies are to be renewed. All ablutions with soap and water, &c. are inadmissible. If the purulent secretion should be too profuse, it may be gently removed with dry lint or soft old linen."

*American Archives of Med. and Surg. Science. No.* 5, *February,* 1835.

---

*Case of Viper's-bite.* By Dr. Siebergundi, of Dorsten.

A woman, while working in a wood, of a sudden experienced an acute pain in the ring-finger of her right hand, as if stung by a bee: on examining the finger, she discovered two small wounds, from which a little blood trickled; and on looking to the ground, saw a grey snake coiled up at her feet. She felt alarmed and distressed; a cold shivering came over her, followed by urgent thirst, vomiting, and inclination to void the fæces. She was conveyed home; and these symptoms continuing, a ligature was applied round the arm, with a view to prevent the venom reaching the heart, and a little honey smeared over the wounds.

At Dr. S.'s first visit, he found the right upper extremity, as far as two hands' breadth above the elbow, swollen, as if ready to burst; the tumefaction was bounded superiorly by means of a red woollen garter, which had been fastened tightly round the arm; the hand and fingers were much swollen, more especially the dorsum; as were likewise the integuments of the wrist-joint, and the forearm. On the third phalanx of the ring-finger, nigh to the articulation, he observed, on both radial and ulnary sides of its volar aspect, a roundish penetrating wound, as if produced by a small obtuse instrument; the wounds were bloodless, and emitted neither serum or other fluid; they were not more sensible to the touch than the rest of the hand. The whole extremity was painful if touched, and of a pale hue; so that the intumescence bore some resemblance to œdema, although the tension present prevented its pitting on pressure. The general symptoms were as follows:—countenance pale, continued rigors, pulse unusually slow and weak; the vomiting had discontinued about an hour before. Resolvent poultices with vinegar to be applied over the arm, and pledgets of charpie smeared over with digestive salve to the wounds. Rad. Serpentar. internally.

The swelling having augmented towards evening, she was ordered, in addition to the above, oleaginous frictions to the borders of the swelling, and a nitrated emulsion internally.

On the next day, the 27th, she was reported to have passed a sleepless night; the swelling had extended to the breast and shoulder, and along the inner side of the upper arm; numerous vesicles had made their appearance, filled with clear serum, but having a dark brown base. The fever was moderate, and no pain of the head was felt. Dec. Chinæ c. Sp. Minder. internally—Dec. Chinæ c. Sacch. Saturni externally, in form of poultice.

On the 28th, soon after having employed the last-named remedies, the pain abated in a great measure, the fever subsided, the surface becoming cool, and the pulse slow and languid. No appetite for food; bowels confined since the receipt of the injury. The finger wounds were found to be cicatrized. The swelling still persisted, and was greatest on the right side of the back and right breast, which had the same volume as in the most active period of lactation: in the subclavicular and deltoid regions several dark blue spots were observable, the result of sugillation.

Along the whole inner and some parts of the outer surface of the upper arm, the epidermis was detached as in sphacelus, the subjacent parts appearing throughout of a dark-blue tint; the remainder of the swollen parts were pale, and painful on being touched, except at the boundaries with the sound skin.

On the 30th, the general symptoms had vanished; the tumefaction had progressively diminished, and the excoriated portions exhibited healthy granulations. A slight œdema of the legs had supervened. From this period is to be dated her convalescence. For some time after her recovery, she complained of a sense of numbness in the right hand, most marked in cold weather; the hand continued paler than natural.

The history of the case now related confirms what has been advanced by MM. Lery and Gräve, in the Allg. Nationalzeit. f. Deutsch, No. 281, viz. that the bite of a serpent, when it involves a part of the body of narrow circumference, as a finger or toe, gives rise to more serious consequences than when an extremity of the body is concerned; for, in the latter instance, the empoisoned fang, grazing over a broad surface, merely frets the skin: whereas, in the former case, the tooth is permitted to penetrate more deeply into the substance, and convey the venom into direct contact with the lacerated tissues.

The general affection, as the feeling of uneasiness and distress, pallor, and chilliness of the surface, vomiting, looseness, and feeling of universal debility, and the local intumescence, together with the bloody extravasation and excoriations around it, are to be ascribed to the bio-chemical agency of the energetic septic animal poison on the circulating mass.—*Heidelberger Klinische Annalen*, Bd. x. H. 3, 1835.

---

### *Hernia of the Appendix Cæci Vermiformis.* By Dr. TARAMELLI.

ON April 19th, 1835, a man, 68 years of age, was admitted into the hospital of Milan, with the symptoms of strangulated inguinal hernia of the right side. He had been ruptured for many years, but the present symptoms had existed about two days. The tumour was the size of a hen's egg, hard and painful; abdomen tense; obstinate constipation; small, contracted, and febrile pulse; no vomiting. Reduction was attempted by the taxis, bleeding, and the warm bath; but in vain, and the operation was decided on. On exposing the contents of the sac, they were found to consist of the appendix cæci vermiformis alone, increased to four times its natural volume. After Dr. Taramelli had divided the neck of the sac and the ring, he gently drew outwards the intestine, in order to satisfy himself more fully of its consisting of the appendix only: the cæcum immediately presented itself, and an indentation exactly at the junction of the cæcum with the appendix, which marked the seat of the strangulation, and showed that the cæcum itself was not involved. The intestines were then returned into the abdomen. An oily aperient procured several stools, and for two days there were no unpleasant symptoms; but a violent fever, accompanied with acute pains, then came on, and an inflammatory swelling of the whole of the side corresponding to the hernia, which, notwithstanding eight bleedings from the arm and forty leeches, terminated in profuse suppuration: this gradually diminished, and ceased about the end of May.

[Surgical writers have recorded cases in which the appendix cæci vermiformis, together with the cæcum, had been contained in a hernial sac; but they make no mention of the appendix alone having been found strangulated.]

*Annali universali di Medicina, vol.* 75, *Fasc. di Sett.* 1835, p. 430.

---

### *On the Treatment to be adopted after the Reduction of Dislocations.* By J. F. MALGAIGNE, Professeur agrégé à la Faculté de Médecine.

TREATISES on distortions are deficient in directions as to the treatment to be adopted after these accidents have been reduced, and, to fill up this hiatus, M. Malgaigne has written a paper on the subject, which was read before the Academy of Medicine. The practical deductions which he has laid down are, that forty days are at least necessary to consolidate the torn articular capsules and ligaments, and in many cases a still longer period: in dislocations of the lower limbs especially, the patients should not walk for sixty days. Simple repose at the commencement is not sufficient, even in those dislocations which appear to remain *in situ*, by themselves. Thus, when the femur has ruptured its capsule upwards and outwards, the position must differ from a rupture downwards and inwards. In the first case the thigh should be extended, and the foot turned outwards; in the second, the foot should be turned inwards. If the bone is dislocated on the pubis, the thigh should be strongly flexed. The rules generally laid down by surgeons are in reference to the prevention of an anchylosis; but there is another object to be fulfilled by position, which is to adjust it so that the ruptured edges of the ligaments should be in contact. In luxations of ginglymoid joints, the most favorable position is demiflexion; for in this attitude the points of insertion of the torn lateral ligaments are the least separated from each other. When an old dislocation is reduced, five or six months are necessary to the cure. In unreduced dislocations of more than two months' standing, where the new capsule is probably becoming organized, and particularly when a change of the articular surfaces is feared, the limb should be held in position by a suitable apparatus until the swelling has disappeared; after which, slight motion in fit directions may be permitted, and the joint strengthened by douche baths, or external cauterization.

*Journal Hebdomadaire de Médecine, No.* 46, 14 *Novembre*, 1835.

---

*New Mode of Treating Luxations of the Sternal End of the Clavicle.*
By M. Velpeau.

No luxation is more easily reduced, or with more difficulty retained in its place. The difficulty is owing to the endeavour of the surgeon to oppose the power which tends to carry the clavicle forwards, instead of relaxing the muscles whose action oppose his wishes. Anatomy and experience show that in these cases the anterior sterno-clavicular and inter-clavicular ligaments are broken, and that dislocation necessarily follows, as the articular surfaces are so constructed that they do not offer any resistance to the muscles, which immediately displace the end of the bone: the pectoralis major and internal portion of the deltoid drawing it forward, the trapezius and sterno-cleido-mastoideus drawing it upwards; whilst both sets are assisted by the large muscles of the trunk attached to the scapulæ. The deltoid, pectoralis major, sterno-cleido-mastoideus, and trapezius are relaxed, and the rest neutralized, by carrying the elbow inwards and forwards to the lower part of the sternum, so that the hand may rest upon the opposite shoulder: when this is done, the bone returns to its place. It can be retained there by the following apparatus: A towel, folded thrice, is first placed round the thorax, and retained by braces attached to its upper border. The arm is then placed in the necessary position, and held by an assistant. The surgeon fixes the end of a bandage (eight yards in length) under the armpit of the sound side; brings it behind over the luxated clavicle; then downwards over the part of the arm; passes it behind and beneath the elbow; then again under the sound armpit, behind the chest, above and before the injured clavicle, so as to make three or four turns like the first. Next he passes the bandage, which has just embraced the elbow, over the fore-arm, then on the sound clavicle, between the hand and the neck, instead of passing it under the armpit as before; after which he passes it downwards behind the thorax, towards the elbow, bringing it again on the fore-arm and clavicle, and making thus three or four diagonal turns. After this the bandage is not again passed under the elbow, but it is passed two or three times from below upwards round the chest and the bent arm; the remaining part is expended by making a few turns like the first, fixed by two or three circular ones. The whole is fixed by pins, and secured by a napkin covering all. This bandage may remain a month or more without any displacement. A more simple contrivance is a belt, or band, passing round the body, and having in front a deep pouch for the elbow. When the belt is fixed, and the elbow placed in the pouch, the belt must be drawn forcibly upwards towards the clavicles, and fixed there by braces over the shoulders, which are attached to the upper border of the pouch, as well as to the edges of the body-belt. *Journal Hebdomadaire de Médecine, Mai* 30, 1835.

### *On the Seat and Diagnosis of Dislocations of the Shoulder Joints.* By B. M. MALGAIGNE.

It is commonly admitted that the head of the humerus may be displaced *downwards*, beneath the glenoid cavity, so as to rest on the neck of the scapula; *forwards and inwards;* and *backwards* into the fossa sub-spinata; besides which, there may be incomplete and consecutive luxations, the latter from muscular action. M. Malgaigne partially dissents from these commonly received opinions, on grounds both of anatomy and practice. The glenoid cavity is surrounded superiorly, anteriorly, and posteriorly by an osteo-fibrous arch (acromio-coracoid), which, as it descends lower behind than before, renders posterior dislocations more difficult than anterior, and, as it is deficient inferiorly, appears to favour luxations in this direction. The scapulo-humeral capsule, however, opposes this, for, although very loose, it is not sufficiently so for any dislocation, except a very partial one forwards, to take place without its being ruptured, at least in half its circumference; and even when the inferior three-fourths are torn, the upper fourth still remaining prevents the head of the humerus from being luxated downwards, as has been commonly imagined, and it is then almost necessarily placed under the coracoid process. The deductions which M. Malgaigne draws from anatomy are—

1. Cæteris paribus, the dislocation beneath the coracoid process is the most easy.
2. This luxation may take place without the capsule being torn, but then it is incomplete.
3. In every complete luxation of any kind the capsule is ruptured.
4. It is impossible that the head of the humerus can be placed on the neck of the scapula, fossa sub-scapularis, or fossa sub-spinata, unless the capsule is torn in all or almost all its circumference, except in those cases where it is unnaturally elongated.
5. In every luxation the arm is found to be elongated, if measured when brought near to the trunk.

In M. Malgaigne's experiments on the dead body, he luxated the head of the humerus beneath the coracoid process, so that the head was freed from the capsule, and directed forwards and inwards; the greater tubercle resting in the inferior part of the glenoid cavity, and the neck applied to the anterior border of the glenoid cavity. This dislocation presented all the symptoms of the luxation downwards of authors, besides some other symptoms not previously described, such as—1. Elongation of the anterior wall of the axilla, measured from the clavicle to the free border of the axilla. 2. Projection of the luxated head forwards, beneath the pectoralis major. 3. Rotation of the humerus outwards, in some cases extreme, in others hardly or not perceptible. From these data three inferences are drawn: 1. Symptoms, such as abduction of the arm, which have been attributed to muscular action, depend on the resistance of the capsule, as they are found in the dead body. 2. The supposed dislocation downwards is only a sub-coracoid dislocation. 3. Important signs of these dislocations had been overlooked. M. Malgaigne has confirmed these conclusions, by proving that the same symptoms are found in dislocations during life, and by showing, from examinations on the dead body, that these symptoms are produced by the sub-coracoid dislocation. Many cases are collected; two of these are striking. The first was in a woman, and Dupuytren (whose diagnosis was proverbially sure,) had pronounced it to be fracture of the neck of the humerus: the application of M. Malgaigne's principles of diagnosis made him alter his opinion, and the dislocation was reduced. The second case supports M. M.'s opinion, that no consecutive dislocations are produced by muscular action. On examining the arm of a Russian soldier which was dislocated at six years of age, and unreduced, the pressure of the edge of the glenoid cavity on the neck of the humerus was found to have been so great as to have made a large and deep depression, dividing the head from the shaft in the same way as in the femur, (nova acta physica-medica.) M. Malgaigne has met with only one decided case in which the dislocation was downwards. This is mentioned by Dessault. The capsule was entirely separated, for the head of the humerus was so moveable that it could be carried with equal ease against the external border of the pectoralis major, against the anterior edge of the latissimus dorsi, and against the skin of the axilla, which are very different from the common symptoms of luxation. The situation of the head of the bone varies a little even in the sub-coracoid dislocation, but without altering its general character. Generally it is found immediately beneath the coracoid process, from which it is only separated by some fibres of the sub-scapularis: sometimes, however, it is 2, 3, 4, or more lines beneath it; at other times it is carried a little more inwardly. Dislocation inwards of authors is called by M. Malgaigne luxation sub-scapularis, and his opinions are new. The arm in this dislocation is longer than the other, but the contrary is the usual opinion. The head of the humerus cannot be felt in the axilla; it projects beneath the clavicle more internally than the coracoid process; the projection is irregular, and formed by the tubercles of the humerus, as is proved by the touch and the direction of the condyles. The arm, instead of being separated from the body, to which it cannot be approximated, as in the sub-coracoid dislocation, is forcibly applied to the trunk, from which it cannot be separated unless by considerable force and pain. From many examinations after death it appears that the head is placed in such cases in the fossa sub-scapularis. M. Malgaigne thinks that partial luxation of the humerus may take place, and that in such cases the head of the bone is always beneath the coracoid process, but not wholly driven out of the glenoid cavity. He therefore does not agree with Sir Astley Cooper, who considers that the head of the bone, in partial dislocation, is situated externally to the coracoid process; and, in criticising Sir Astley's cases, shows that in the cadaveric post-mortem observations, the head of the bone is stated to be beneath the coracoid process. This may take place without rupture of the capsule, but it is generally torn. In three cases the arm could be approximated to the trunk, but this does not seem to be constant. The arm itself is elongated. This is a more common dislocation than is supposed. It is a singular fact that the polished spherical head of the humerus resting on the polished edge of the glenoid cavity is not moved by the muscles, and may remain there until a deep groove is formed. Sir A. Cooper and Dupuytren agree that it is difficult to reduce and to maintain in its position afterwards. M. Malgaigne has remarked that the pain in partial dislocations is greater than in others, which he attributes to the capsule being more stretched because it is less torn. It is generally caused by force acting directly on the shoulder.

[This memoir elicited a discussion in the Academy of Medicine, and subsequently a letter from M. Malgaigne. The important point which M. Malgaigne wishes to establish is, that *in every luxation of the humerus the arm is elongated.* M. Velpeau objects to this; but it appears that sufficient care was not taken to measure the arms accurately in the cases which he adduces as exceptions. The arm in all cases should be approximated, as much as possible, to the trunk, and the distance measured between the inferior angle of the acromion and the external condyle. M. Lisfranc, although disposed to agree with the author, makes the sensible remark, that the words "always and never" should be erased from pathology. The practical value of the fact that the arm is invariably elongated in dislocation, is seen in its application to the diagnosis between this accident and fracture of the neck of the humerus, which is often difficult. In only two cases (according to M. Lisfranc,) can the arm be elongated when three is fracture of the neck, viz. with paralysis of the limb, or with oblique fracture, when, by extension, the upper end of the lower fragment has been brougnt to rest upon the lower end of the upper portion. These two accidents, however, are rare, and easily recognised, and in all others this sign is of great diagnostic value.]

*Gazette Médicale, Oct.* 1835. Nos. 40, 41.

---

*Observations on the Employment of the Suction Pump in Incarcerated Hernia.*
By Dr. L. Koehler, of Warsaw.

In 1828 Dr. K. succeeded in reducing a scrotal hernia, after it had remained obstinately incarcerated for three days, by the merely incidental application of a cupping glass. Having, then, never heard of the employment of the suction-pump in similar cases, he scarcely ventured to attribute his success to the real cause: yet the occurrence impressed itself upon his mind, and induced him, on reading Dr. Busch's Observations on the use of the Suction-pump, in Hufeland's Journal, for 1832, to give this new method of reducing incarcerated herniæ a fair trial. The first experiment was made on a man, aged sixty, who had been affected with a scrotal hernia of the left side for nine years, and which suddenly became incarcerated, in consequence of an indigestion. The patient became affected with violent colic, and all attempts on the part of a surgeon to effect reposition were fruitless. When Dr. K. first saw him, he had been in this state for three days; and the scrotal sac had attained the size of an ostrich's egg. The features were sunken, and indicated great anxiety; the body suffused with a cold sweat; abdomen inflated, hard; extremities cold; pulse scarcely perceptible, and thready; bowels not relieved for three days; fæcal vomiting and singultus ensued. In vain the most active treatment had been already employed, and an immediate operation appeared to be the only possible means of saving the patient, although, under the circumstances, even that resource seemed next to hopeless. The swollen and inflamed parts being still capable of bearing some degree of pressure, Dr. K. made an ultimate attempt at taxis; but that failing, the suction-pump was then had recourse to. On its first application over the abdominal ring, borborygmi were heard within the incarcerated sac, and, to the surprise and satisfaction of every one, reposition was effected immediately afterwards. The vomiting instantly ceased, a few hours later the bowels acted freely, and the patient entirely recovered in a few days.

Six cases of incarcerated inguinal and one of incarcerated crural hernia (in a female) are further detailed, in all of which the strangulation, after resisting every other kind of treatment, short of actual operation, yielded almost immediately to the relaxing powers of the suction-pump.

In the instance of a Jew pedlar, however, greater perseverance in the new method was found requisite. This individual (whose age was sixty-two) had been affected, during a period of twelve years, with a scrotal hernia, which, owing to a mismanagement of the truss, became ultimately strangulated. When the patient was first examined at the Jewish hospital, the hernial sac was of the size of a child's head, and somewhat tense, though not remarkably tender. The patient had vomited twice, but his bowels had (in spite of several clysmata, which were administered to him before entering the hospital) remained constipated for four days. No success having attended a first application of the suction-pump, venesection and an anodyne ointment were had recourse to, together with the exhibition of calomel in liberal doses. No improvement was, however, effected by these means; the vomiting returned, the fever continued unabated, whilst the affected part become more and more painful. Under these circumstances, Dr. K. resolved on renewing the application of the suction-pump. This second attempt again proved a failure; but on the trial being repeated, slight peristaltic motion within the hernial sac became perceptible, and immediately after the fourth application of the instrument, complete reposition of the hernia was effected without difficulty. Copious alvine evacuations ensued shortly afterwards, and in a few days the individual had recovered his former health.

In an obstinate case of strangulated inguinal hernia, a colleague of Dr. K. once succeeded in accomplishing his purpose of reduction, by simply applying an inverted tumbler, by way of a cupping-glass, at the point of strangulation.

Professor Janikowski communicated to Dr. Koehler a case of strangulated umbilical hernia, wherein he had employed the suction-pump with perfect success. The patient, a corpulent unmarried woman, aged fifty, had been afflicted with umbilical hernia, which never admitted of perfect reposition for the space of two years, when true symptoms of strangulation supervened. After three days of obstinate costiveness, stercoraceous vomiting ensued. The hernial protuberance was of the size of a large orange, hard and very sensitive. All the usual remedies having proved abortive, the suction-pump was at length resorted to. Its application was productive of great pain, and the integuments became, to a certain extent, excoriated, as they drew up and filled the glass bell. The instrument, having been allowed to operate for some little time, was at length withdrawn, when *complete* reduction of the hernia was effected with perfect ease. Abundant stools quickly led to recovery; and the application of a suitable truss removed every inconvenience under which the woman had formerly laboured.

A further enumeration of cases in which taxis was accomplished, and the knife superseded by means of the suction-pump, appears to Dr. K. to be superfluous. In twenty-three cases, most of which were of a hopeless kind, the new method never once failed him; and it were greatly to be wished, he concludes, that so simple a remedy might be called into more general use.

*Hecker's Annalen. Erster Band. Viertes Heft*, 1835.

